# On Public Disinfecting Stations and the Use of Steam under Pressure as a Disinfectant
*Part of Report of Section on Sanitary Science made to the Medical and Chirurgical Faculty of Maryland, April 27th, 1892.


**Published:** 1893-03

**Authors:** Eugene F. Cordell

**Affiliations:** Professor of Principles and Practice of Medicine, Woman's Medical College of Baltimore.


ARTICLE II.

### ON PUBLIC DISINFECTING STATIONS AND THE USE OF STEAM UNDER PRESSURE AS A DISINFECTANT.*

BY EUGENE F. CORDELL, M. D.,

Professor of Principles and Practice of Medicine, Woman's Medical College of Baltimore.

With the increase in our knowledge of micro-organisms and the accumulating evidences of their wide-spread and baneful influence, there has been a corresponding increase in the attention paid to disinfection and in the realization of its supreme importance. Indeed, it may be said that the prevention and treatment of disease have largely resolved themselves into the discovery of agents which will destroy these insidious and deadly enemies of our health and lives, and yet be harmless to ourselves and not injurious to our property. One of the most important advances made in preventive medicine of recent years has been the passage of the law requiring notification of infectious diseases, and I desire, in passing, to commend the determination evinced by our health commissioner, Dr. McShane, to enforce this law—so essential to the health of our people and to the welfare of this community. By its aid alone can prompt measures be adopted for isolating the sick and disinfecting infected localities and articles.

There is no difference of opinion and none in principle, though details vary, as to the use of disinfectants in rooms which have been occupied by those sick of infectious diseases and for their contents. I have no intention of opening up a discussion of disinfectants generally, and the

---

* Part of Report of Section on Sanitary Science made to the Medical and Chirurgical Faculty of Maryland, April 27th, 1892.

limits of this brief paper would not permit me to do so. I will only say that a thorough scrubbing of floor and wood-work with soft soap, scraping, if necessary, and white-washing the walls, hot white-wash perhaps being preferable to cold, fumigating the room with sulphur and soaking the linen, etc., in disinfectants and then prolonged boiling, are effective measures in proportion to the thoroughness and intelligence with which they are carried out. According to Rubner (who is Koch's successor in the chair of hygiene in the University of Berlin), all germs ar : destroyed by boiling in water for one-half hour; it requires twenty minutes to destroy the tubercle bacilli, ten minutes for the typhoid bacillus, and simply exposure to boiling water to destroy those of cholera and diphtheria. This must, therefore, be regarded as a mode of disinfecdon to be preferred whenever it can be employed.

But apart from the element of uncertainty as to thoroughness and the difficulties in the way in attempting to carry out such measures in private apartments, there are articles, such as mattresses, carpets, hangings, heavy clothing, etc., which could not, owing to their bulk, or other causes, be treated by them, or which might be injured by them. This leads us to consider what other measures are available.

Realizing our needs in this direction, most of the German cities (and it is to Germany that we look at this time for leadership in this field) have established central disinfecting stations, where furniture, bedding, etc., are subjected at public cost to the most approved modern methods of disinfection. England and France and other European countries have adopted the same measure, and some of our large cities are awakening to its importance. I deem it quite unnecessary to argue at length in favor of such a service in a city of the size and importance of this, with the authorities wide awake to all the advances in municipal government of the day, provided only the facts are duly appreciated. I shall endeavor briefly to describe what has been learned and done regarding this most important subject.

It has long been known that heat surpasses all other agents as a disinfectant. At first it was supposed that a dry heat equal to that of boiling water would destroy all germs of disease. Then it was found that there were organisms which could withstand even a higher temperature than this—as the spore-bearing organisms, of which the anthrax bacillus was taken as a representative: it not being known at that time that the spores of this organism are exceptionally resistant, and that most other organisms—the tubercular, typhoid, diphtheritic, choleraic, erysipelatous, and others—are far less tenacious of life. Even sulphurous acid, as we have learned, is only effective when applied in connection with steam, and yet this combination is particularly injurious to clothing and other fabrics. Dry heat is likewise injurious and does not penetrate through heavy materials. Steam heat is much less harmful than either and can be applied to the disinfection of most materials, especially if they be first heated to the boiling point by dry heat and dried again subsequently to the steaming. To Koch himself we owe the discovery of the great germicidal power of steam. According to all researches hitherto made, steam under pressure is the ideal disinfectant. No known form of life, either animal or vegetable, can survive a brief exposure to its influence. On this point all authorities are agreed.

What are the essential points, then, to be observed in the construction of a public disinfecting station? Such a station should be centrally and conveniently located, on ground isolated from dwellings and frequented places, and enclosed by a high wall. No other business should be allowed on or near the disinfecting works. The establishment should be under the control and management of the city authorities, or if the service be given out by contract, it should be strictly understood that no other business is to be carried on in connection with it, such as carpet cleaning, etc. Th[illegible]ould also be frequent and thorough inspection of the [illegible]mises and procedure by a competent person. Separate vehicles and drivers should be provided for conveying articles from houses to the station; the drivers should be dressed in some distinctive garb, and the vehicles should be stabled to themselves. These vehicles should be air-tight and the articles should be placed in bags and taken through a window to them, not carried through the passage and hall-way. Such articles should be submitted to fumigation before removal from the house. After disinfection they should be returned to the owner by a separate conveyance and they should never be returned to infected apartments. The disinfecting building should be divided into two compartments by a brick partition, into which should be built the steaming chamber or stove. It should have a door of entrance on one side of this partition and a door of exit on the other side, so that materials once disinfected are not by any possibility brought in danger of reinfection.

The heating apparatus employed is usually cylindrical in shape. It is heated by a furnace or by Bunsen burners or gas-jets. The dome should be peaked or angular, so the steam condensing on its roof may run down the sides and not drip on to the clothing, etc. Steam is admitted by means of a pipe. It should always be introduced at the top of the stove and allowed to escape from the bottom into the opposite apartment. This downward current against gravity is considered very important, because the steam thus finds its way into every crevice and nook of the stove, which it might not do if it came in at the bottom and escaped at the top. The apparatus should be air-tight and should be lined inside with asbestos to avoid injury from contact of linen, etc., with the metal sides. The articles to be disinfected should lie on frames in the centre, or be hung up. Musty bedding and old clothes should not be introduced with new articles and fine linens, as the smell of the former, which is not destroyed by heat, will be communicated to the latter and remain attached to them for a long time.

Having introduced the articles to be disinfected, the temperature of the chamber is raised to the boiling point (212° F.), so that the steam shall not condense on cold surfaces. The steam is then let in and continuously applied for a period of twenty minutes, which is long enough for ordinary purposes, or for thirty minutes at the maximum. The time element is of great importance because too long an exposure will injure certain materials. The temperature is assured by means of a thermometer, placed inside the bundle of clothing, etc., and communicating with a battery on the outside; by a simple device a bell may be made to ring when the desired temperature is reached; or a thermometer may be placed in the tube by which the steam escapes. V. Budde (*Zeitschrift fur Hygiene*, Band VII, p. 269) deprecates excessive heating of the steam, as that interferes with condensation, which permits the powerful ef fect of the latent heat to be brought to bear upon the materials. Excessive heat is also objectionable, as stated above, on account of the possible injury to the articles. There is some difference of views as to the degree of pressure to which the steam should be subjected. Some of the most approved apparatus used allows of a pressure as high as 25 pounds to the square inch. The London *Lancet*, in the "Report of its Special Sanitary Commission on Disinfecting in London," January, 1891, refers with strong approval to the establishment of Mr. W. G. Lacy, formerly medical assistant at a London fever hospital, who disinfects for a large part of the city of London and for a great number of private houses, schools, etc., within a radius of twenty or more miles around London. The stove here employed is of great strength and the huge, thick, heavy door, which is screwed down, bulges out under the force of the high pressure. There is a safety-valve to prevent explosions. Even exposure to this tremendous pressure is not considered sufficient by Mr. Lacy, since it only compresses the air in the thickness of mattresses, etc., and the moist heat will not penetrate these bubbles of compressed air. To break up these bubbles they are made to expand rapidly by letting off some of the steam and then turning on again more steam. This is the most powerful process of disinfecting known, for whereas dry heat and sulphurous acid, whether dry or moist, exert only a superficial effect,steam under high pressure will penetrate the heaviest mattresses and even more compact substances. Scientific experiments have shown that germs placed in the centre of a thick mattress are killed in fifteen minutes by it (*Lancet*, loc. cit.) Into such an apparatus it is not necessary to introduce separate articles, to open mattresses, etc.; the articles are put in canvas bags before being taken out of the infected room and introduced into the oven without unpacking. In consequence, a greater number of articles can be disinfected each time and the process is conducted with great rapidity. The stove of Mr. Lacy is exceptionally large, having a capacity of five cwt. The degree of pressure is determined by a simple manometer. Many consider that a pressure much below the above is sufficient—say two pounds to the inch. The President of this Society, Prof. Welch, tells me h[illegible]s of this opinion, and in view of the great expense [illegible] risk connected with the high pressure apparatus, this opinion is entitled to careful consideration.

Mr. Lacy has also a stove for articles not requiring penetration, or susceptible to injury, as boots, books, mantel-piece ornaments, spring-mattresses, etc., and for articles that can be spread out so as to expose all their contents.

It cannot be denied that the establishment of such an institution involves great expense and requires a large force of specially trained employees; moreover, to conduct it requires a man of scientific qualifications—it certainly will not fulfill the purpose desired if entrusted to an ignorant or untrained person. Its advantages are incalculable, and when we contemplate it in the light of modern knowledge and discovery it becomes simply a necessity. Certainly, should an epidemic arise in our midst—at any time a possibility—we should be in but a poor plight without it.

One important point is to be constantly borne in mind in order to make this or any other mode of public disinfection feasible, and this is, that the materials must be returned to their owners uninjured. Nothing will deter people from sending their clothes, etc., to be disinfected so much as to have them once sent back in a damaged condition. Some discrimination is necessary, therefore, in deciding what articles can be subjected to steaming; leather, stamped plush, skins and water-proof materials cannot; linen or similar materials will be iron-moulded if brought in contact with iron; grease should be removed beforehand, if possible, otherwise it becomes fluid and is liable to spread; blood and pus are coagulated and can be afterwards removed by chlorine or other solvents. According to Levison, fast-dyed materials are not injured. A lady's seal-skin jacket is not damaged by 240° F. Intermittent heating below 212° F. is advised for kid gloves. Othe[illegible]etails regarding this part of the subject are given by Lev[illegible]*Zeitschrift fur Hygiene*, Band VI, p. 225). Finally[illegible] suggestion has been made and it carries with it its own recommedation—that there should be some place provided where poor people may go whilst their houses and clothing are being disinfected.

It may be well, in concluding, to consider what is being done in other cities, especially of our own country.

In Boston, in "scarlet fever and diptheria," reliance is placed upon burning sulphur, nothing is said of other diseases. There is a building at the quarantine station where steam disinfection is practised. I have not learned further details.

In New York, disinfection is practised by dry sulphur fumigation; beds, pillows, mattresses, upholstered furniture, etc., being cut open and contents spread out. Bedding or other removable articles are taken to the disinfecting depot on East River, where they are "thoroughly disinfected by hot air or steam, in an elaborate apparatus, especially constructed for that purpose."

In Washington City, the room where a patient has been ill with scarlet fever or diphtheria is thoroughly disinfected by exposure for several hours to the fumes of chlorine gas, or of burning sulphur. All clothing, bedding, carpets and other textiles which have been exposed to the contagion are required to be either burned, exposed to super-heated steam, or thoroughly boiled.

Fumigation with sulphur is practised, and it is stated that "the owners of a private establishment for the renovation of carpets, bedding and other textiles, have constructed, in an isolated place in the District, a separate tank for the cleansing of articles that have been exposed to infection, by super-heated steam and naphtha."

At the quarantine stations in Charleston and New Orleans, steam heat is employed and the methods and apparatus seem to be very thorough.

In London the methods vary; the city authorities prefer sulphur fumigation, but the use of steam-disinfection is growing and the large stove of Mr. Lacy above mentioned "disinfected for one district alone more than nine tons of articles during the Christmas quarter." Thc district of St. Pancras has built for its use a stove which is an exact copy of Mr. Lacy's. A number of districts employ Lyons' steam pressure stove, which is much smaller, so that two are sometimes required.

In Germany, there is more uniformity in practice, and the principles which I have endeavored to explain and recommend are there carried out with the highest degree of efficiency. A useful addition to the so-called refuges in Berlin is the bath which can be taken by persons whilst waiting to have their clothing disinfected in the adjoining steam-chamber. In Cologne, for economic reasons and to give employment to stokers and others temporarily out of work, the apparatus is connected with the steam laundry of the city hospital. The schimmel apparatus seems to be very popular in Germany.

In Paris there are three public disinfecting ovens, and the service is carried on with all the precautions I have narrated above.

Moscow as yet has only a sulphur fumigating establishment. I might give further details collected in my investigation of the subject did time permit.

In this city the measures pursued for disinfecting apartments and their contents correspond with those usually employed, but in a circular entitled "Suggestions for Preventing the Spread of Scarlet Fever," issued by the Board of Health, I find the following: "Most articles may be disinfected in a room if hung up loosely and exposed to the fumes of sulphur, though it would be an additional safeguard to expose anything thick, like a bed-mattress, to prolonged heat at a temperature of about 240° F.; and indeed, *heat* or steam must, with our present knowledge, be considered *the best disinfectant*." (Italics in original.) That our present efficient Health Commissioner is alive to the importance of the considerations which I have urged above, appears from the fact that last fall he secured permission from the city authorities for the erection of a frame building for fumigation. This building adjoins the morgue and is under charge of the keeper of the morgue. It consists of two layers of boarding filled in between by four inches of saw-dust, so as to be as air-tight as possible, and has a capacity sufficient to admit a hack. About three pounds of sulphur are used for each thousand cubic feet of space. It has been in operation now since about the middle of November and a large quantity of bedding and bed-clothes have been disinfected there. Twelve to fourteen hours are deemed sufficient time for exposure of the articles to the sulphur fumes. While from the facts presented above this arrangement cannot be considered sufficient, it is a step in the right direction and will doubtless lead in no great while to more efficient methods.

